# Development of Compound Fault Diagnosis System for Gearbox Based on Convolutional Neural Network

**DOI:** 10.3390/s20216169

**Published:** 2020-10-29

**Authors:** Ming-Chang Lin, Po-Yu Han, Yi-Hua Fan, Chih-Hung G. Li

**Affiliations:** 1 Department of Mechanical Engineering, Chung Yuan Christian University, Taoyuan 32023, Taiwan; patience19960212@gmail.com (P.-Y.H.); yihuafan@cycu.edu.tw (Y.-H.F.); 2 Graduate Institute of Manufacturing Technology, National Taipei University of Technology, Taipei 10608, Taiwan; cL4e@ntut.edu.tw

**Keywords:** gearbox, convolutional neural network, accelerometers, remote fault diagnosis, convolution kernels

## Abstract

Gear transmission is widely used in mechanical equipment. In practice, if the gearbox is damaged, it not only affects the yield rate but also damages other parts of machines; thus, increases the cost and difficulty of maintenance. With the advancement of technology, the concept of unmanned factories has been proposed; an automatic diagnosis system for the health management of gearboxes becomes necessary. In this paper, a compound fault diagnosis system for the gearbox based on convolutional neural network (CNN) is developed. Specifically, three-axis vibration signals measured by accelerometers are used as the input of the one-dimensional CNN; the detection of the existence and type of the fault is directly output. In testing, the model achieved nearly 100% accuracy on the fault samples we captured. Experimental evidence also shows that the frequency-domain data can provide better diagnostic results than the time-domain data due to the stable characteristics in the frequency spectrum. For practical usage, we demonstrated a remote fault diagnosis system through a local area network on an embedded platform. Furthermore, optimization of convolution kernels was also investigated. When moderately reducing the number of convolution kernels, it does not affect the diagnostic accuracy but greatly reduces the training time of the model.

## 1. Introduction

In modern society, various kinds of products are produced by machines where the gear mechanism relies on the teeth on the rim to mesh with other gears to transmit torque. Due to its high transmission efficiency, accurate transmission ratio, large power range, and the ability to change the speed or direction of movement, gears are widely used in machinery. In recent years, with the advancement of technology and the concept of unmanned factories, the use of machines to replace workers can not only reduce labor costs but also reduce human errors and dangers caused by long hours of work. Thus, it increases the production rate and stability.

The research on the fault diagnosis of the rotor system can be traced back to Randall [1] where some common types of gear faults and frequency spectrum characteristics were introduced. Generally, the accelerometers or laser vibrometers were the most used for signal detection; and the spectrum distribution of the signal can be interpreted by the experienced technicians to determine the type of fault. However, human judgments sometimes make uncertain errors. In recent years, with the progress of science and technology, the computing speed of computers has increased dramatically, so some machine learning methods that require high-speed computing, such as convolutional neural networks (CNN), support vector machines (SVM) [2], etc., once again attract everyone’s attention. Comparing CNN and SVM, the classification accuracy of CNN would be higher than that of SVM, especially for the complex systems like the compound fault diagnosis of rotating machinery. Therefore, the goal of this paper is to develop a CNN model to achieve the fault diagnosis of a gearbox; and with the combination of the local area network, the fault diagnosis can be predicted from the remote site.

The artificial neural networks (ANN) was first inspired by Hebbian theory [3] proposed by Donald Olding Hebb, and after Rumelhart et al. [4] proposed the back-propagation method to automatically correct the weights of the multilayer perceptron (MLP), the ANN method became concrete and began to be widely used. Although the multilayer perceptron relies on its huge internal variables and nonlinear functions to handle highly complex nonlinear problems, it cannot effectively learn the relationship between spatial data. And, this issue was effectively improved when LeCun et al. [5] proposed CNN approach. Furthermore, Krizhevsky et al. [6] used CNN method for image recognition to win the championship in the ImageNet competition held in 2012. After that, CNN related research began to flourish in various fields, including object recognition [7,8], portrait recognition [9,10], action recognition [11,12], etc. In addition to the use of the above-mentioned imaging field, neural networks are also used for fault diagnosis in the mechanical field. Chen et al. [13] used graph of vibration signal as input of CNN network for fault diagnosis of gearboxes. Jia et al. [14] used the frequency-domain signal, converted from fast Fourier transform (FFT), as input to the neural network for fault diagnosis of bearings and planetary gearboxes. The weighting matrix of the network was corrected by a specific way. In addition, Janssens et al. [15] and Jing et al. [16] also used frequency-domain signal as input to CNN for machine diagnosis. They all showed the spectrum signal was suitable for the extraction of fault characteristics. Zhang et al. [17] used WDCNN (wide first-layer kernels deep CNN) method for fault diagnosis. In the first convolutional layer, they used a larger kernel for noise attenuation and used AdaBN (adaptive batch normalize) skill to improve its robustness. Zhao et al. [18] converted the data into 2D images through wavelet-transform and used DRN (deep residual network) to diagnose faults. Furthermore, a dynamic adjustment layer was used, which can be automatically corrected during training process to find out the more obvious frequency characteristics. In contrast to accelerometer-based diagnosis, the motor encoder signal was used in the work of Jiao et al. [19]. With some signal processing for obtaining angular speed and acceleration signals, the stitching data are fed to the one dimension CNN for fault diagnosis. Wu et al. [20] used the raw vibration data as input of one-dimensional CNN for fault diagnosis of rotating machinery and much better performance than traditional methods was shown. Liang et al. [21] converted the vibration data into a two-dimensional picture through wavelet-transform to perform compound fault diagnosis. The disadvantage of this method is that the conversion of 2D images would increase the computational cost during CNN training, and it will also increase the computational time during actual diagnosis.

Through the literature survey, it can be found that CNN architecture is widely adopted and can get good results, but most of them are focus on a single fault. For the above reasons, here a simple and compound diagnosis method is investigated where the accelerometers are used to capture the vibration signals of the 3-axes, and a one-dimensional CNN is used for the compound fault diagnosis of the gearbox. The main differences between this work and the previous ones are the comparison of various input data types and the optimization of the convolution kernel size which are investigated in Section 3.

## 2. Brief of CNN

**Figure 1:**
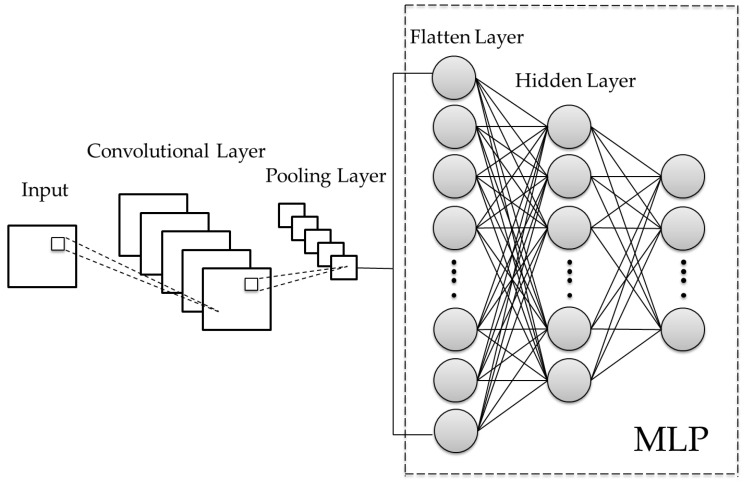
Convolutional neural network (CNN) architecture diagram.

The main difference between CNN and traditional MLP lies in the addition of the convolutional layer and the pooling layer as shown in Figure 1. The emergence of CNN has made rapid progress in machine learning in the visual part. It mainly uses the concept of mask in a machine-vision study where convolution operations are performed in the convolutional layer, and the amount of calculation can be reduced through the introduction of the pooling layer. Finally, a large number of complex neuron connections in MLP are used to assist the machine in classification and learning [22,23]. The calculation method is described below.

### 2.1. Convolutional Layer

The convolutional layer contains several convolution kernels. Each convolution kernel will perform convolution operations on the partial area of the input data, and then add a bias term and get the output data after the activation function. Convolution operation can help extract different features of the input image such as edges or lines, etc., which is why CNN is good at handling graphics problems. Here, $K_{i}^{l}\;$and$\;b_{i}^{l}$ are used to represent the *i*th convolution kernel and bias term in the *l* layer, respectively, and $x^{l}\left(j\right)$ represents the *j*th part of the input data in the *l* layer. g represents the activation function, $y_{i}^{l}\left(j\right)$ is the output result of the *i*th convolution kernel for the *j*th partial data in the *l* layer, so the calculation equation of the convolution layer is as follows:

(1) $$y_{i}^{l+1}\left(j\right)=g\left\{K_{i}^{l}*x^{l}\left(j\right)+b_{i}^{l}\right\}$$

### 2.2. Pooling Layer

The pooling layer is generally set after the convolutional layer. The pooling layer can retain the output characteristics of the convolutional layer while reducing the size of the output data, thereby reducing the overall calculation of the CNN. The most commonly used method of the pooling layer is Max-pooling, which uses the largest value in the area data as the representative output, and the calculation method of the maximum pooling is as follows:

(2) $$P_{i}^{l+1}\left(j\right)=\underset{\left(j-1\right)W+1\leq t\leq jW}{\max}\left\{q_{i}^{l}\left(t\right)\right\}$$

where $q_{i}^{l}\left(t\right)$ represents the *t*th data point in the output of the *i*th convolution kernel in the *l*th layer, *W* represents the pooled area, and$\;P_{i}^{l+1}\left(j\right)$ represents the output value of layer *l* + 1.

### 2.3. Flatten Layer

The flatten layer is an important design to connect pooling layer and MLP. Its function is to convert the output data after the convolution operation into a one-dimensional form before inputting the data into the MLP.

### 2.4. Multilayer Perceptron

**Figure 2:**
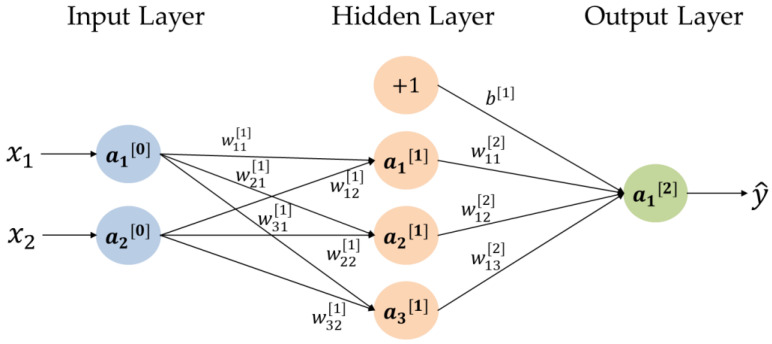
Multilayer perceptron with dual inputs, a single hidden layer and a single output.

Multilayer perceptron consists of three parts, i.e., input layer, hidden layer and output layer as shown in Figure 2. The calculation method of neuron $a^{\left[l\right]}$ of layer *l* is as follows:

(3) $$a^{\left[0\right]}=\left[\begin{array}{c} x_{1}\\ x_{2} \end{array}\right]$$

(4) $$w^{\left[1\right]}=\left[\begin{array}{cc} w_{11}^{\left[1\right]} & w_{12}^{\left[1\right]}\\ w_{21}^{\left[1\right]} & w_{22}^{\left[1\right]}\\ w_{31}^{\left[1\right]} & w_{32}^{\left[1\right]} \end{array}\right]$$

(5) $$z^{\left[1\right]}=w^{\left[1\right]}\cdot a^{\left[0\right]}$$

(6) $$a^{\left[1\right]}=g^{\left[1\right]}\left(z^{\left[1\right]}\right)$$

(7) $$z^{\left[2\right]}=w^{\left[2\right]}\cdot a^{\left[1\right]}+b^{\left[1\right]}$$

(8) $$a^{\left[2\right]}=g^{\left[2\right]}\left(z^{\left[2\right]}\right)=\hat{y}$$

where w represents the weighting matrix, *b* represents the bias term, and g represents the activation function.

### 2.5. CNN Architecture of This Study

**Figure 3:**
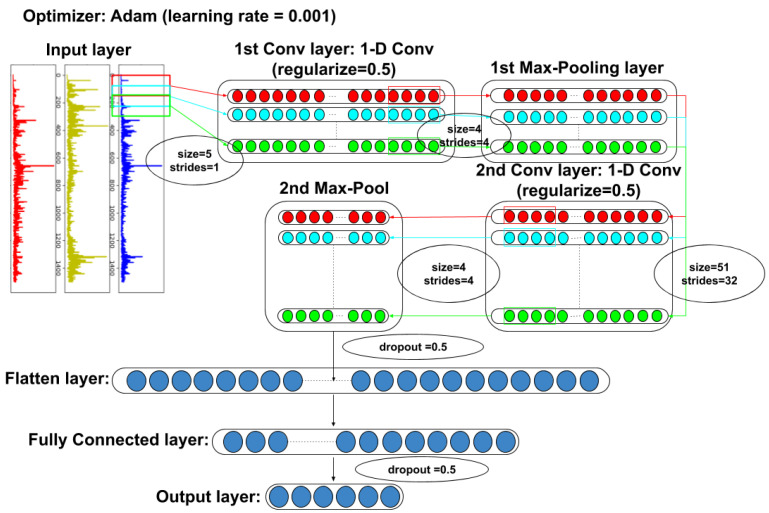
The CNN architecture of this study.

In this study, the adopted CNN architecture refers to the work of Jiao et al. [19], and modifies the size of the convolution kernel of the convolutional layer. Here, the detailed number of parameters used in CNN and the architecture of the CNN are shown in Table 1 and Figure 3:

## 3. Experimental Study

### 3.1. Description of Experimental System

**Figure 4:**
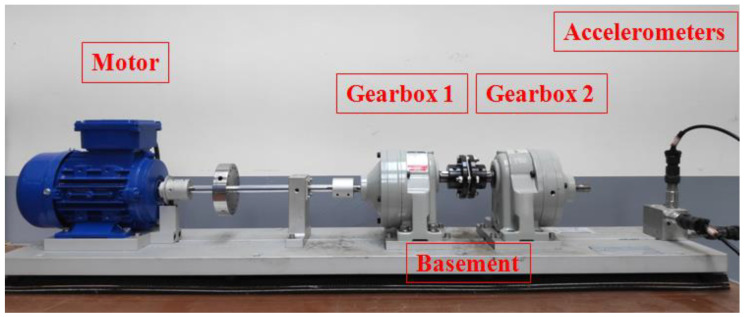
Experimental platform of helical gearbox driven by motor.

**Figure 5:**
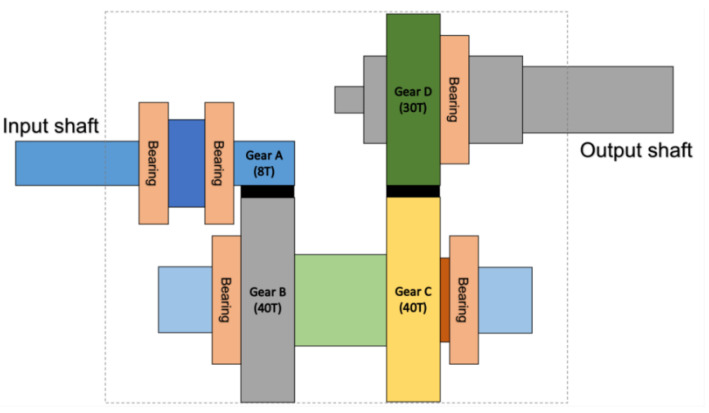
Gearbox structure.

The experiment in this study uses a servo motor to drive helical gearboxes as shown in Figure 4 where three accelerometers are mounted on the basement to capture the vibration waveform of the gearbox. The material of the basement is aluminum and the size is about 1000 mm × 200 mm × 25 mm. In addition, the structure of the gearbox is shown in Figure 5. Here, the system development is based on Python 3.7.6, and Tensorflow 2.1 is used to construct CNN. The CPU of the computer used for network training is i7-8700, memory is 24 GB, and GPU is NVIDIA RTX 2060 (6 GB). The sampling frequency of the vibration signal is 10 kHz and the time span of sampling is 1 s. The captured signals are then fed into CNN model for classification of faults.

**Figure 6:**
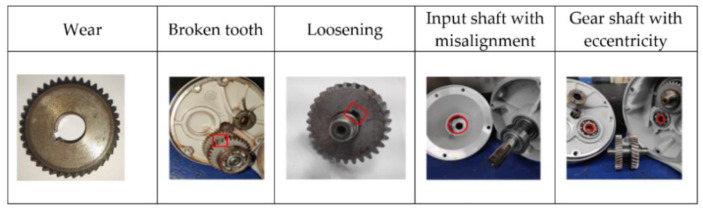
Gearbox with 5 different faults.

In this study, five types of gearboxes with different faults were produced by hand-made processing, including wear, broken teeth, loosening, misalignment of the input shaft, and eccentricity of the gear shaft, as shown in Figure 6.

Various faults are described as follows:

- Wear: wear gear C and gear D by carborundum;
- Broken tooth: grind out one tooth of gear C;
- Loosening: no key is installed between gear and shaft, but they are tightly matched to make it possible to slip;
- Input shaft with misalignment: enlarge the input shaft bearing seat by 0.3 mm, and pad the input shaft so that it is not concentric with the shaft of gear A;
- Gear shaft with eccentricity: enlarge the bearing inner diameter of gear B and gear Cby 2 mm.

**Figure 7:**
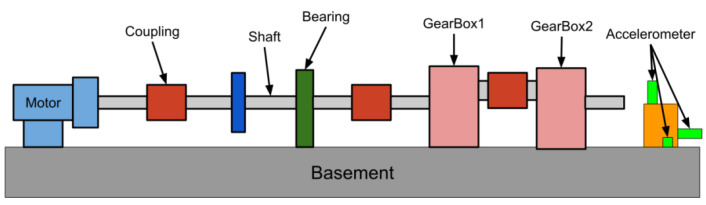
Schematic diagram of composite fault vibration data capture.

In order to diagnose compound faults, we use a coupling to connect two faulty gearboxes to simulate the compound fault as shown in Figure 7. For data acquisition, it is divided into three kinds of speeds of 1800, 2400 and 3000 rpm, and 500 data records for each speed. And the ratio of the training set to test set is 7:3, that is, in each fault combination, the training set has 350 data records, and the test set has 150 data records.

**Figure 8:**
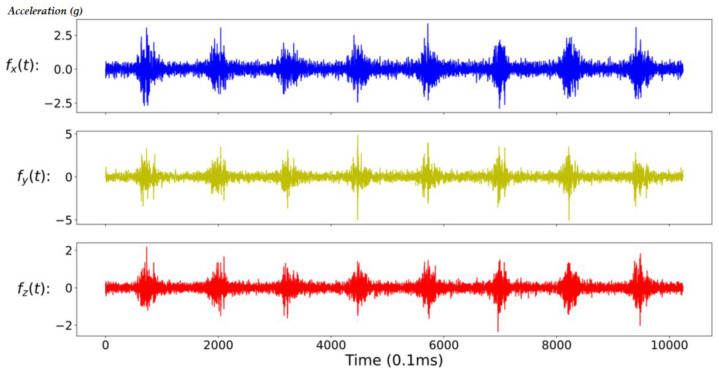
Full-time (FT) data.

**Figure 9:**
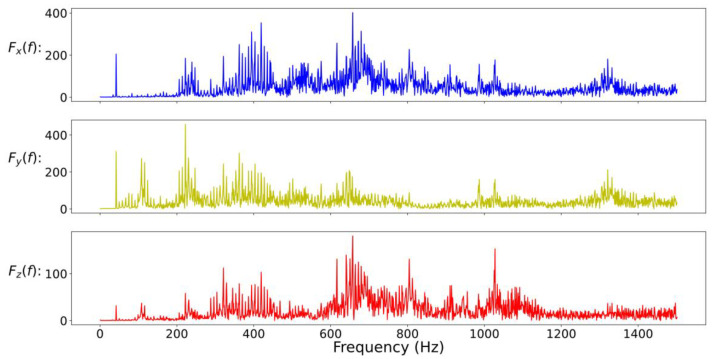
Low-frequency (LF) data.

**Figure 10:**
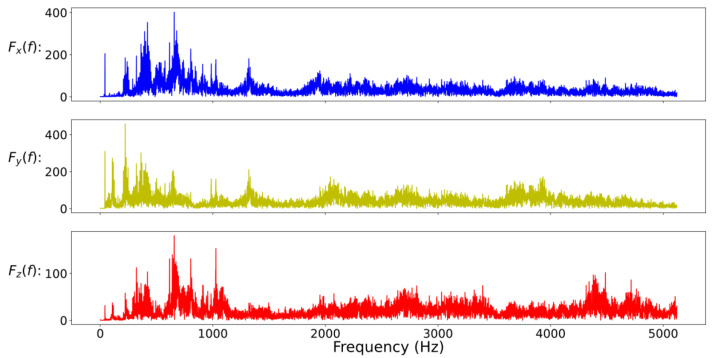
Full-frequency (FF) data.

**Figure 11:**
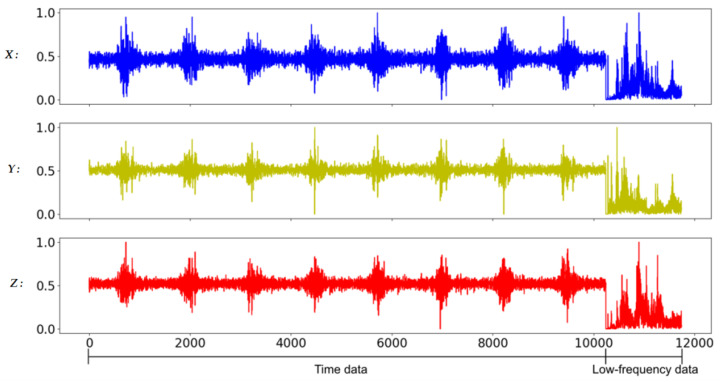
Full-time combining low-frequency (FTCLF) data with normalization.

**Figure 12:**
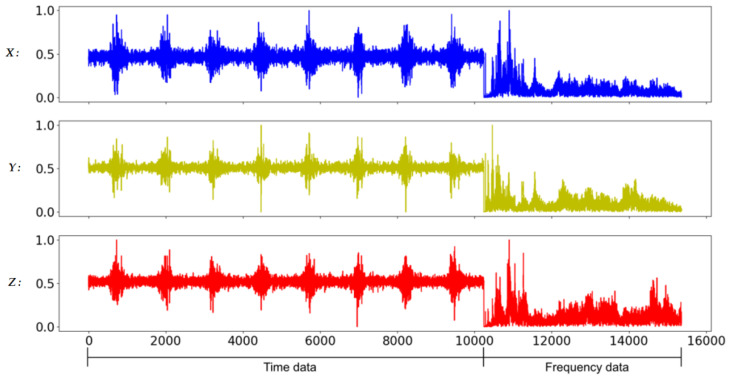
Full-time combining full-frequency (FTCFF) data with normalization.

In general, the characteristics of the fault may appear in the time domain or the frequency domain. In this study, in addition to the time-domain and the frequency-domain data as the training input data of the CNN model, the time-domain and frequency-domain data are also combined as the new training input data. Due to the large difference in amplitude between time-domain data and frequency-domain data, it will be normalized before splicing. In addition, in mechanical applications, high-frequency data is often filtered out because it is considered to contain a lot of noise. Therefore, this study also compares the results of filtered and unfiltered data; so, here will cover five data formats, namely full-time (FT) data (as shown in Figure 8), low-frequency (LF) data (as shown in Figure 9), full-frequency (FF) data (as shown in Figure 10), full-time combining low-frequency (FTCLF) data with normalization (as shown in Figure 11), and full-time combining full-frequency (FTCFF) data with normalization (as shown in Figure 12). Here, the waveforms in Figure 8, Figure 9, Figure 10, Figure 11 and Figure 12 were captured under the condition of wear with broken-tooth.

### 3.2. Remote Diagnosis

**Figure 13:**
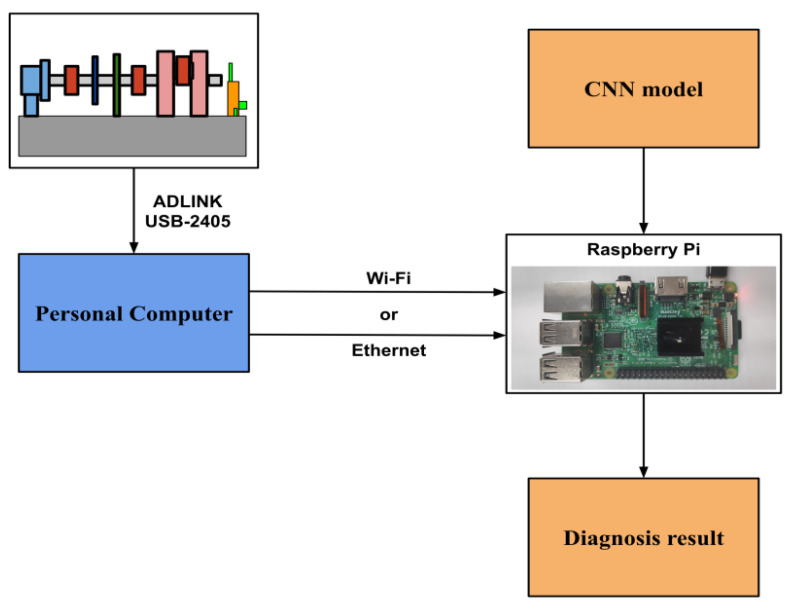
Schematic diagram of the diagnosis process.

In a factory, multiple gearboxes are often used in the transmission mechanism, and the gearboxes may be scattered widely. Usually, an accelerometer is placed next to the gearbox to measure the vibration signal. In order to facilitate the collection of signals, wireless transmission can be used. The remote site will build a receiving center to collect the signals and perform individual fault diagnosis; then, the result can be uploaded to the control center. Thus, it can realize instant fault detection on the production line. In order to verify this approach, the platform of Raspberry Pi [24] is used for remote fault diagnosis. When the training of the CNN model is completed, the model will be stored in the Raspberry Pi and the receiving program will be executed. When the new vibration data is captured, the data will be sent to the Raspberry Pi via Wi-Fi by the “Socket” API function of the computer. And Raspberry Pi will perform pre-processing such as FFT conversion of the vibration data, and then the data are fed into the CNN model for diagnosis, and then the diagnosis results are displayed. Here, the diagnosis process is shown in Figure 13:

From Figure 13, the personal computer is the client, and the Raspberry Pi is the server. In the server, the input of the port number, the location of the vibration data, and the selected CNN model are required. When the data is transmitted to Raspberry Pi, the diagnosis is executed; then the result will be displayed on the screen of Raspberry Pi when the calculation is complete. It only takes about 1 to 2 s for each data to be transmitted and diagnosed.

### 3.3. Experimental Results

Here, the five different fault gearboxes described in Section 3.1 and a normal gearbox are used to perform a compound fault diagnosis. Considering the operating conditions of combining two gearboxes (as shown in Figure 7), there are 15 different combinations. And two situations under compound faults are discussed as follows.

#### 3.3.1. Case (a): Comparison of Various Input Data Types and a Different Number of Neurons in a Fully Connected Layer

**Figure 14:**
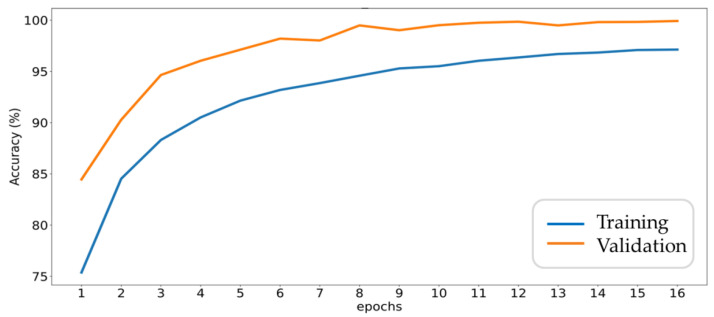
Accuracy trends during the training and validation process.

**Figure 15:**
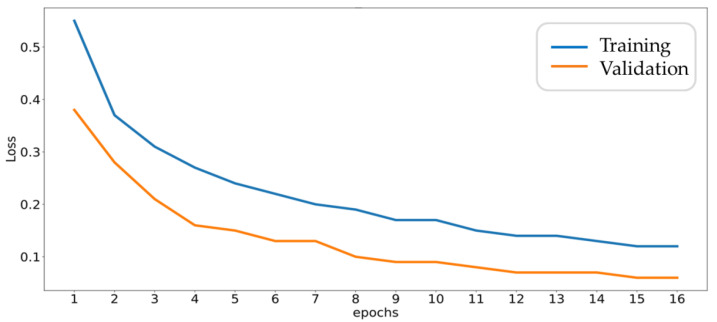
Loss trends during the training and validation process.

Since the initial random value of parameters in the training process will affect the results of the CNN, this study will train each model 10 times, and use a different random sequence for each training, thereby reducing the impact of randomness. Then, the model will be represented by the best performance among the 10 training results, and the hyperparameters are shown in Table 2:

As described in Section 3.1, 30% of data records are reserved for test or validation which are not used in model training. The overall accuracy and loss trends with each epoch for training set and cross-validation set are shown in Figure 14 and Figure 15, respectively, where FF data are used as input and 240 neurons are adopted in fully connected layer. From Figure 14, it can be seen that the accuracy of the training set gradually increases with successive training, while the accuracy of the cross-validation set follows the trend and eventually approaches 100%. Furthermore, from Figure 14, the accuracy gap between the two curves is related to the dropout mechanism. Here, due to the software configuration of library, the dropout enabled in the training process would reduce the accuracy. On the contrary, the dropout is turned off in the validation process. Another reason for the gap is the fault signals of gearboxes are not diverse enough due to limited fault samples.

**Figure 16:**
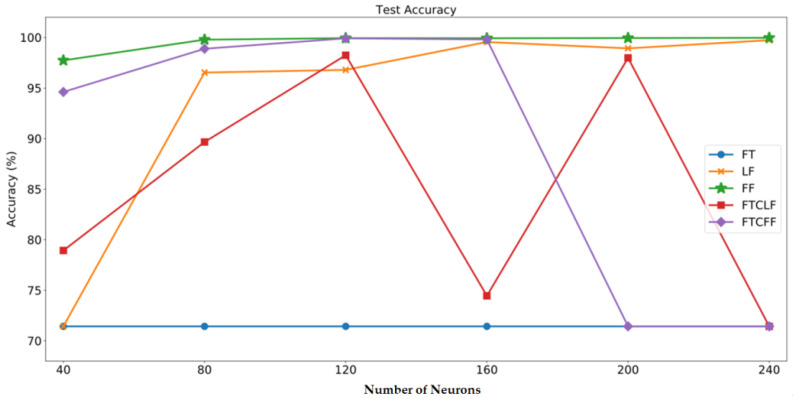
Accuracy results of the test set.

In order to find the most suitable number of neurons in the fully connected layer, the number of neurons in the fully connected layer in this study starts from 40, and increases by 40 each time till to 240. After the training, the accuracy results of the test set are shown in Table 3 and Figure 16:

From Figure 16, it is obvious that the accuracy result of the FT case is the worst, on the other hand, the FF data type can obtain the best result. This is because the starting point of the time-sequential data is not fixed, this implies the characteristics of the time-domain data is not stable. Since CNN is not good at handling sequential problems, it is hard to obtain sufficient and stable features from the time-domain data, so the accuracy is the lowest. On the other hand, the frequency-domain data can be obtained by converting time-domain data through FFT. In contrast, since the frequency-domain data is not dependent on the starting point of each sampling, so it can provide more stable characteristics than the time-domain data. Therefore, the diagnostic accuracy in FF case is the best. For the same reason, the results of FTCLF and FTCFF are worse than FF can also be explained by the usage of time-domain data in FTCLF and FTCFF would deteriorate the characteristic information.

Next, comparing the cases of FF and LF, it can be found that the FF method performs better than the LF method, indicating that the high-frequency signal would contain the characteristic of the fault. If the high-frequency signal is discarded, it will naturally affect the accuracy of its diagnosis.

Then, comparing the results of different amounts of neurons in the fully connected layer, it can be found that a moderate number of neurons can get a more stable result. If 40 neurons are used, the neural network will not be able to effectively respond to various inputs due to insufficient neurons. If more neurons like 240, there may also be a risk of overfitting.

#### 3.3.2. Case (b): Optimization of the Number of Convolution Kernels

From Table 1, it can be seen that the numbers of convolution kernels used in the first and second convolution layers are 16 and 32, respectively. When we observe the output of the pooling layer, we find that only a few convolution kernels have outputs, and the others are almost zero. This may indicate that the scale of this CNN is too large for this diagnostic system, resulting in only a small number of variables are used; thus, many convolution kernels are corrected to approaching zero during the training process. A more suitable number of convolution kernels can be adjusted by observing the output of the convolution kernel. After checking the output value, we can find that only two convolution kernels (1st, 13th) are required in the first convolutional layer and only one convolution kernel (5th) is required in the second convolutional layer. Therefore, after changing the number of convolution kernels in the two convolutional layers, the number of internal variables in the model can be greatly reduced, as shown in Table 4.

**Figure 17:**
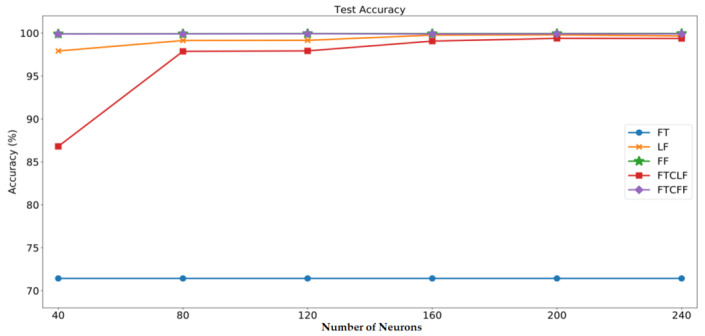
Accuracy results of the test set.

After the revised CNN model is trained, the accuracy results of the test set are shown in Table 5 and Figure 17:

Comparing Figure 16 and Figure 17, it can be seen that the overall performance in revised CNN has been significantly improved. It is due to the chance of each variable of the revised model being trapped in the local minimum during training is reduced. Except for the case of FT data type, when the number of neurons in the fully connected layer is 80 or more, the diagnostic accuracy rate of each model can reach more than 95% where the accuracy of FF or FTCFF cases is almost 100%.

In order to compare the training time before and after the revision, each model is trained for 2 epochs and the required time is recorded as shown in Table 6:

It can be seen from Table 6 that the training time of the revised model can be effectively reduced by 27.42%.

## 4. Conclusions

In this paper, a remote compound fault diagnosis system for gearbox is developed where one-dimensional CNN is used. In testing, the model achieved nearly 100% accuracy on the fault samples we captured. The main contributions of this work are the comparison of various input data types and the optimization of the convolution kernel size. In our results, the frequency-domain data can provide better results of diagnostic accuracy than the time-domain data because of stable characteristics in the frequency spectrum. Furthermore, it also shows a high-frequency signal retains useful information for fault diagnosis. In addition, appropriate selection of the kernel size can not only reduce training time but also improve accuracy. For practical usage, a measured vibration signal can be transmitted via a local area network. Ideally, it only takes about 1 to 2 s for data transportation and processing; thus, achieves rapid diagnosis. Finally, it should be noted that the amount of fault gearboxes used in this paper is limited; in actual applications, if more data on the spot can be used to increase the diversity of training data, the neural network model will be more complete.

## Figures and Tables

**Table 1:** Number of parameters used in the CNN.

No. Layer	Layer Type	Kernel Size (Stride)	Kernel Number	Output Size
1	Convolution	5 × 1 (1)	16	5116 × 16
2	Max Pooling	4 × 1 (4)	16	1279 × 16
3	Convolution	51 × 1 (1)	32	1229 × 32
4	Max Pooling	4 × 1 (4)	32	307 × 32
5	Dropout	0.5	/	307 × 32
6	Flatten	/	/	9824
7	Fully connected	200	/	200
8	Dropout	0.5	/	200
9	Output	6	/	6
Total parameters:	2310345			

**Table 2:** Parameter values used in the training model.

Training times	10
Epochs each time	200
Training/Test batch size	50
Training iteration per epoch	441
Test iteration per epoch	189

**Table 3:** Accuracy results of the test set (content: accuracy).

	Number of Neurons					
Input	40	80	120	160	200	240
FT (%)	71.43	71.43	71.43	71.43	71.43	71.43
LF (%)	92.27	98.60	99.90	99.85	97.97	97.70
FF (%)	95.10	99.95	99.93	99.93	99.94	99.92
FTCLF (%)	71.43	95.28	98.25	99.60	98.96	71.43
FTCFF (%)	99.45	99.93	99.91	99.95	99.92	99.92

**Table 4:** Number of variables before and after convolution kernel correction.

	Revised Model	Model before Revision
Variable quantity	75501	2385846
Variable reduction	2310345	
Variable reduction (%)	96.83	

**Table 5:** Accuracy results of the test set (content: accuracy).

	Number of Neurons					
Input	40	80	120	160	200	240
FT (%)	71.43	71.43	71.43	71.43	71.43	71.43
LF (%)	97.92	99.14	99.16	99.77	99.80	99.67
FF (%)	99.92	99.93	99.95	99.94	99.95	99.96
FTCLF (%)	86.81	97.88	97.93	99.07	99.39	99.37
FTCFF (%)	99.93	99.90	99.93	99.90	99.92	99.91

**Table 6:** Comparison table for training time.

	Revised Model	Model before Revision
Total training time (s)	2160.05	2976.20
Time reduction (s)	816.15	
Time reduction (%)	27.42	
